# The effect of C/N ratio and its frequent addition on commensal and pathogenic bacterial abundances in shrimp *Litopeaneus vanname* gut in a biofloc system: Ratio and frequent addition interaction matters

**DOI:** 10.1371/journal.pone.0283841

**Published:** 2023-04-03

**Authors:** Abdallah Ghonimy, Zhao Chen, Jian Li

**Affiliations:** Key Laboratory of Sustainable Development of Marine Fisheries, Yellow Sea Fisheries Research Institute, Chinese Academy of Fishery Sciences, Qingdao, China; Tanta University, EGYPT, EGYPT

## Abstract

The environmental biotic and abiotic factors form a complicated relationship with the host intestinal microbiota. In our study, we applied different levels of C/N ratio (10, 15, 20) and frequent addition times (once, twice, triple a day) in a factorial experimental design. GC/LC analysis of filtrated biofloc (BF) samples revealed the highest relative fold change for the untargeted bioactive molecules among different treatments, whereas the 16s rRNA analysis revealed the change in the shrimp gut microbiota composition. Based on the available literature on the relationship between the bioactive molecules and the available bacteria in this study, the next bioactive molecules were discussed. Proline was associated with Bacteroidota, Flavobacteriaceae, Gammaproteobacteria, and Flavobacteriales. Plumbagine was associated with Norcardiaceae. Phytosphingosin was associated with Bacteroidota. Phosphocholine compound was associated with Bacteroidota. The monobutyl ether, benzofuran, and piperidone were associated with Micobacteriaceae and Mycobacterium. Generally, C/N 15 and 20 once a day, and C/N 20 triple a day have showed a merit over other treatments in term of low pathogenic and unfavorable bacteria, and high commensal bacterial abundances. The revealed bioactive molecule composition showed the complicity of BF as a source for novel compounds as biosecurity agents in BF system. These molecules could be developed to feed additives upgrading the biosecurity level in aquaculture systems. Other bioactive molecules require future studies to reveal novel molecules in term of aquaculture biosecurity control.

## 1. Introduction

In light of worldwide population growth and increased food demand, the development of the aquaculture industry has been enhanced [1]. Aquaculture rapid development comes with difficulties of degraded water quality and disease outbreak in an intensive farming system [2–4]. These production issues are usually managed by water exchange, which is described for an enormous amount of water drainage and subsequent an environmental impact [5, 6].

Closed aquaculture systems require a low rate of water exchange, and they enhance water treatment and culture biosecurity leading to a low or zero water exchange rate [7]. The clear-water recirculating aquaculture system and biofloc aquaculture system (BF) are the common aquaculture closed systems [7]. By adding carbohydrate substrates to the culture water of BF system, the heterotrophic assimilation activity can rapidly remove the ammonia from water [8], but this bacterial activity generates a load of suspended solids which include a large amount of particulate organic substrates [9–11].

The environmental microbiota influences the intestinal microbiota of shrimp [12, 13], these gut bacterial communities are called an extra “organ” for the aquatic animals as they habitat the digestive tract, that their effect extends beyond the digestive efficiency to the organism’s health and immunity [14, 15]. The intestinal and environmental microbiota interaction is the most complicated relationship between the host and an environment in BF system regarding to its complicated bacterial community compared to other aquaculture systems [16, 17]. The interaction elements including viable microbes and dead bacterial cells including their compartments [18]. For an example, the high relative abundance of Vibrios enriched the number of Vibrios in shrimp intestinal tract in BF system [18]. That the presence of selective pressures leads to the recruitment of specific microbial inhabitants within the shrimp intestinal tract [18]. In fact, shrimp could directly feed on bioflocs [16], these contain different metabolites’ sources [9–11] besides viable microbes and dead bacterial cells. In fact, microorganisms metabolic by-products could play an important role in modulating the intestinal microbial composition as bioactive molecules, this includes quorum sensing agents and N-Acyl-Homoserine lactone bioactive molecule [19, 20]. In the BF system, the carbon addition is a routinely practice which affect the bacterial composition regarding to the carbon source and carbon availability [19–22]. Thus, carbon availability could change the bacterial metabolic by-product composition.

Nutrient availability forms the microbial structure by inducing relevant enzymes and cofactors [23], supplying carbohydrates induces the activity of heterotrophic bacteria in BF system [16]. Since C/N ratios ranged from nine to 12 decrease the denitrifying bacterial abundance [24], whereas C/N ratios ranged from 15 to 18 increase the heterotrophic abundance [25]. Thus, the C:N ratio management influences the bacterial composition in BF system [16]. In term of C/N ratio, this could suggest the effectiveness of frequent addition on bacterial activity, since the added carbon at a moment of addition could represent a specific ratio, for an example, C/N ratio of 15, with a frequent addition of three times, this means a C/N ratio of five at each frequent time of addition. According to our knowledge, one study has investigated the effect of addition timing, not the frequent addition times, of C/N ratio of 15 on daily and weekly basis on the water quality, biofloc volume, growth performance in an African catfish *Clarias gariepinus* biofloc system, among those two-timing treatments, the ammonia and nitrite levels were not significantly different, and the growth was within the proper range, whereas the final biomass was higher in the treatment of daily basis addition [26]. Generally, all those studies have not investigated the effect of C/N ratios and frequent addition interaction on the bacterial composition in a shrimp gut.

To the best of our knowledge, this the first study investigated the effect of C/N ratio and frequent addition interaction on the BF non-targeted molecule composition and intestinal bacterial composition in shrimp. In our study, we applied the GC/LC analysis to reveal the non-targeted molecules composition in BF, and 16s rRNA analysis to reveal the intestinal microbial composition. These could reveal the interaction between environment and shrimp intestinal microbial composition in BF [18]. We hypothesized C/N ratio and frequent addition could affect the non-targeted molecules composition by affecting carbon availability for the bacterial community. This study was expected to provide recommended practices to improve the BF biosecurity, and suggested some molecules as feed additives based on the available literature, in addition to revealing the existence of other molecules which required further studies to reveal novel agents. This information provided a theoretical basis for biosecurity studies aquaculture systems.

## 2. Results

### 2.1. Biofloc size

**Fig 1:**
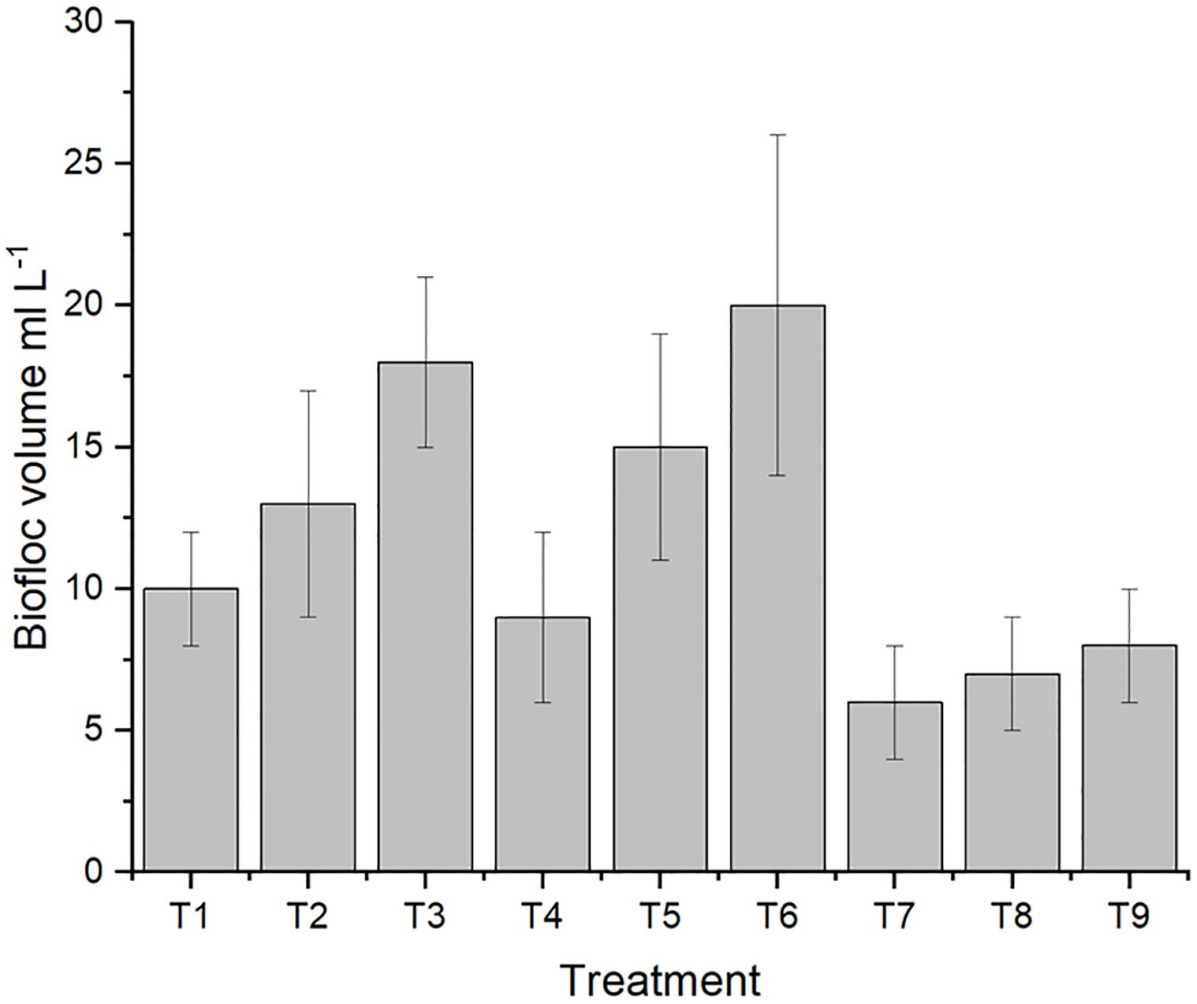
The effect of C/N ratio and frequent addition on biofloc volume. A factorial experimental design included carbon source (starch) frequent addition on daily basis (once, twice, triple) and C/N ratio (10, 15, 20). T1; once C/N 10, T2; once C/N 15, T3; once C/N 20, T4; twice C/N 10, T5; twice C/N 15, T6; twice C/N 20, T7; triple C/N 10, T8; triple C/N 15, T9; triple C/N 20.

Biofloc size was measured by Imhoff cone for the treatments at the beginning of the experiment (1.0) and at the end of the experiment as presented in Fig 1. The highest BF volume was at T3 and T6, whereas the T2 and T9 showed lower BF volume. The treatments T9 and T2 showed a lower pathogenic bacterial abundance as it showed later (section 3). Since high BF volume expresses high heterotrophic bacterial activity, this competes with pathogenic bacteria in space and nutrients. But in this study, the lower BF was not necessary a limiting factor for pathogenic abundances.

### 2.2. Untargeted molecules profile

**Fig 2:**
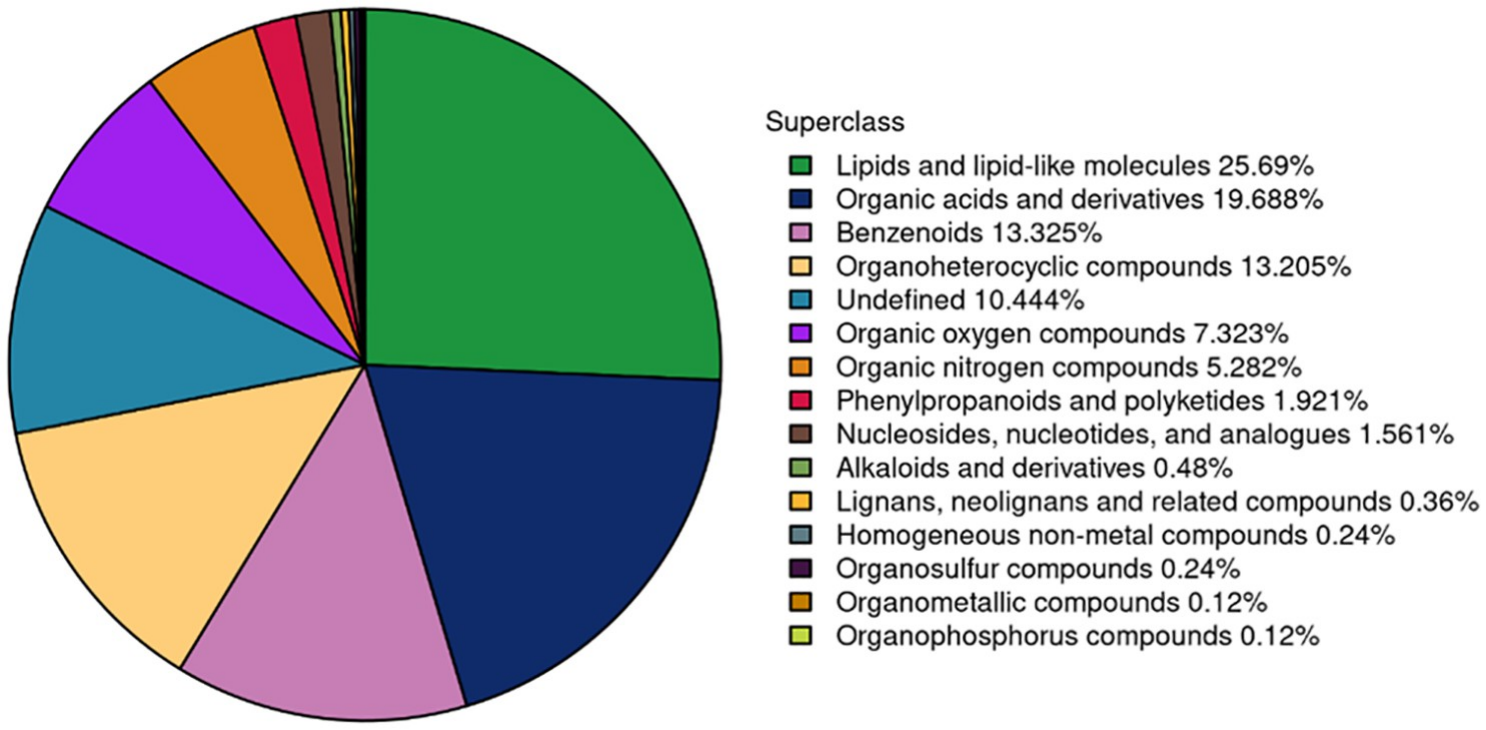
Chemical superclasses of biofloc-derived molecules.

BF is a complicated environment regarding to the accumulated organic molecules from different sources including diet, shrimp metabolic by-products, bacterial metabolic by-products, dead microorganisms, and water xenobiotics. In this study, the most dominant molecules were lipids, organic acids, benzenoids, and organoheterocyclic compounds (Fig 2). A total of 4570 molecules were detected, and a total of 460 molecules were identified, a total of 39 molecules among the identified molecules were nominated as possible active molecules depends on fold-changes, where +2 fold-changes was the lower limit for nomination.

**Fig 3:**
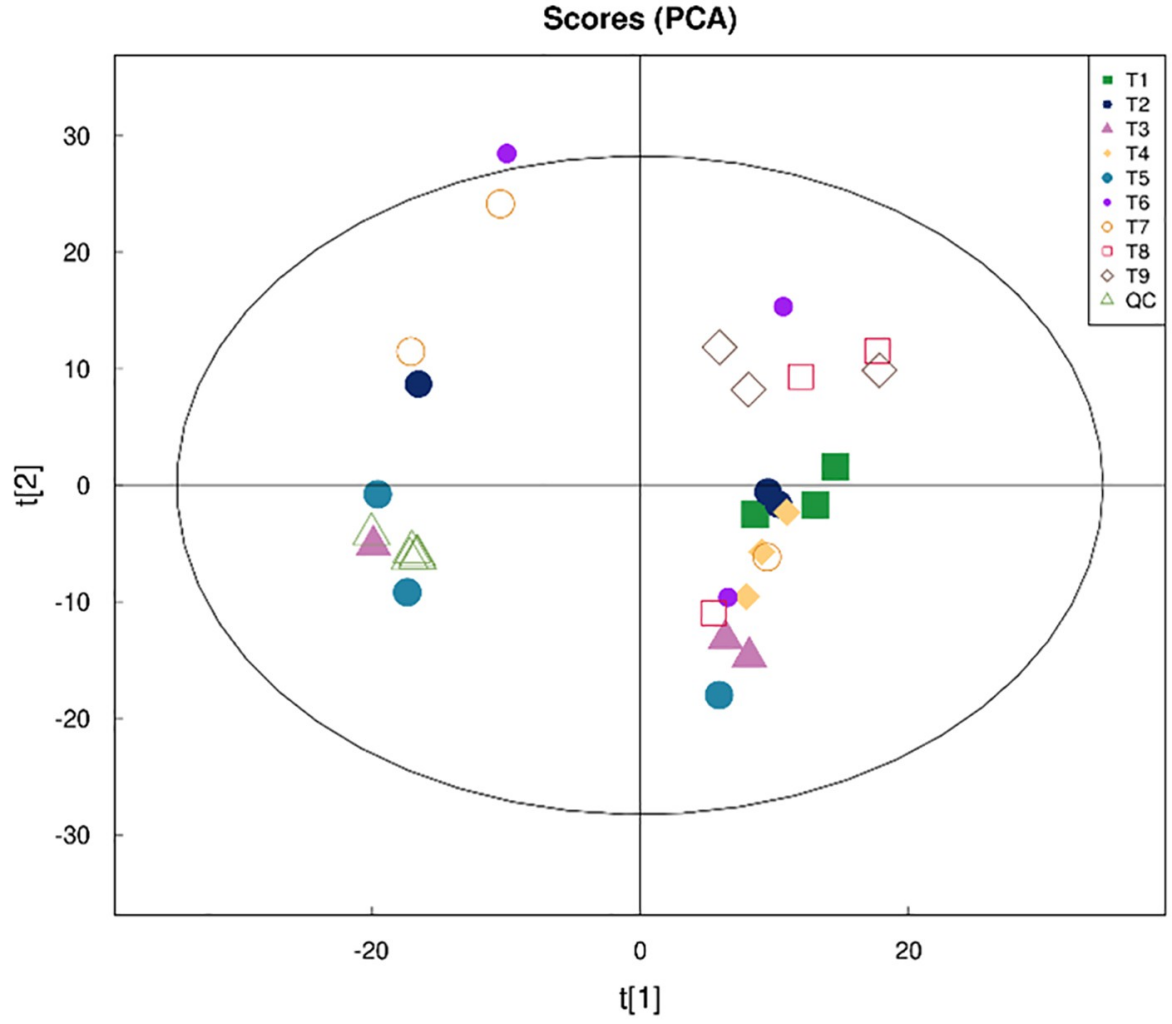
Principle coordinate analysis (PCA). A factorial experimental design included carbon source (starch) frequent addition on daily basis (once, twice, triple) and C/N ratio (10, 15, 20). T1; once C/N 10, T2; once C/N 15, T3; once C/N 20, T4; twice C/N 10, T5; twice C/N 15, T6; twice C/N 20, T7; triple C/N 10, T8; triple C/N 15, T9; triple C/N 20.

Principle coordinate analysis (PCA) showed the distance between treatments, treatments T1, T2 and T4 showed a short distance among each other, whereas treatments T3, T5, T7, T8, and T9 showed a longer distance away from T1 and T2. In fact, treatments T2, T3, and T9 showed a clear distance among each other (Fig 3) as they showed the less pathogen abundances as presented later (section 3). This revealed the bioactive molecules variety among treatments.

#### 2.2.1. Bacterial activity activators

BF showed molecules variety among treatments regarding to the interaction between carbon source (starch) frequent addition and C/N ratios, this included anti-stressors, infection factors, growth factors, and bacterial metabolites (Table 1).

**Table 1:** Biofloc derived molecules as bacterial activity activators.

Treatment*	Molecule name	Associated bacteria	Reference
**T9**	L-proline	Anti-stressor and carbon and nitrogen source for the pathogenic bacteria	[27, 28]
**T9**	Didodecyl 3,3’-thiodipropionate oxide	Carbon source for *Variovorax paradoxus*, this bacterium decreases the activity of *Pseudomonas syringae* by affecting its quorum sensing system	[29, 30]
**T9**	DL-arginine	It is metabolized by *Bacellus subtilis*	[31]
**T9**	1-Stearoyl-2-hydroxy-sn-glycero-3-phosphocholine	It is an factor in *Pneumoccal bacteria* infection	[32]
**T8**	D-alanine	A growth factor for Lactic acid bacteria A metabolite of *Streptococcus faecalis*	[33]
**T6**	Serotonin	Induces the quorum sensing of *Pseudomonas aeruginosa*	[34]
**T6**	M-cresol	A metabolite from tyrosine fermentation by *Clostridium difficle* Decreases the bacterial activity of *E*. *coli Kelbsiella oxytoca Bacteroides thetaiotaomicron*	[35]
**T6**	Triethylene glycol monobutyl ether	A bacterial metabolite from *Mycobacterium xenopi*	[36]
**T5**	1,3-propanediol, 2-amino-2-(2-(3-aizdo-4-octylphenyl)ethyl)	A cross feeding metabolite between *Bifidobacterium breve* and *E*. *coli* (producers) and *Lactobacillus reuteri*	[37]
**T5**	3-hydroxykynurenine	A metabolite from tryptophan degradation which increases the bacterial adherence (SCFA bacteria)	[38, 39]
**T4**	Methylmalonic acid	A metabolite from propionate degradation	[40]
**T4**	3-mehtyl-l-histidine	A substrate for lactic acid bacteria	[41, 42]

* A factorial experimental design included carbon source (starch) frequent addition on daily basis (once, twice, triple) and C/N ratio (10, 15, 20). T1; once C/N 10, T2; once C/N 15, T3; once C/N 20, T4; twice C/N 10, T5; twice C/N 15, T6; twice C/N 20, T7; triple C/N 10, T8; triple C/N 15, T9; triple C/N 20.

#### 2.2.2. Bacterial activity inhibitors

BF showed molecules variety among treatments regarding to the interaction between carbon source (starch) frequent addition and C/N ratios, this included a variety of antibacterial agents (Table 2).

**Table 2:** Biofloc derived molecules as bacterial activity inhibitors.

Treatment*	Molecule name	Associated bacteria	Reference
**T9**	Adenosine	Attenuates bacterial LPS	[43, 44]
**T9**	3-iodo-l-thyronine	It has a bactericidal effect on *E*.*coli*	[45, 46]
**T9**	Plumbagine	Antibacterial agent on *Bacillus subtilis* Inhibits quorum sensing in *P*. *aeruginosa Chromobacterium violaceum Serratia marcescens*	[47–49]
**T9**	Phytosphingosine	Phyto anti-bacterial agent	[50]
**T9**	Stearamide	Phyto anti-bacterial agent against some pathogenic bacteria such as *Pseudomonas putida*	[51, 52]
**T7**	12s-hydroxy-5z,8z,10e,14z-eicosatetraenoic acid	Phyto anti-bacterial agent against *E*. *coli Klebsiella pneumoniae*	[53]
**T7**	19-hydroxyandrost-4-ene-3,17-dione	Anti-bacterial agent	[54]
**T7**	Gelsemine	Anti-bacterial agent against pathogenic bacteria	[55]
**T6**	1,2-dimethyllimidazole	Anti-bacterial agent	[56]
**T5**	1,2-dihydrodesoxymetasone	Anti-bacterial agent	[57]
**T4**	Mehtyl jasmonate	Phyto anti-bacterial agent against *Pseudomonas syringae*	[58]
**T3**	1,9b-dihydroxy-6,6,9a-trimethyl-1,5,5a,7,8,9-hexahydrobenzol(e)(2)benzofuran-3-one	Anti-bacterial agent	[59, 60]
**T3**	2,2,6,6-tetramethyl-4 piperidone	Anti-bacterial agent	[61]

* A factorial experimental design included carbon source (starch) frequent addition on daily basis (once, twice, triple) and C/N ratio (10, 15, 20). T1; once C/N 10, T2; once C/N 15, T3; once C/N 20, T4; twice C/N 10, T5; twice C/N 15, T6; twice C/N 20, T7; triple C/N 10, T8; triple C/N 15, T9; triple C/N 20.

#### 2.2.3. Xenobiotics

BF showed molecules variety among treatments regarding to the interaction between carbon source (starch) frequent addition and C/N ratios, this included drugs, anti-fungi, bacterial substrates, and an insect-repellent agent (Table 3). This molecule composition was differed among treatments, even they have no direct relationship with the bacteria under current study, but they may affect the bacterial composition as they are substrates for some bacteria. Specificity between those xenobiotics and bacterial abundances have not been investigated in literature for those bacteria in current study. Future studies are required to investigate the effect of water contamination on culture microbiome, in addition to the BF bioremediation effect on xenobiotics clearance.

**Table 3:** Biofloc xenobiotics derived molecules.

Treatment*	Molecule name	Associated bacteria	Reference
**T9**	1-palmitoyl-2-linoleoyl-rac-glycerol	Drug	[62]
**T9**	2,3-dihydroxy-3-methylbutyric acid	Drug	—
**T8**	Fenpropidin	Anti-fungi	—
**T7**	Morpholine	A substrate for Mycobacterium	[63]
**T7**	21-hydroxyprogestrone	A substrate for Human microbiota	[64]
**T7**	Megestrol acetate	Appetit inducer	[65]
**T7**	Oxandrolone	Drug	[66]
**T6**	6-methylchromone	Drug	[67]
**T6**	N6-(1-iminoethyl)-l-lysine	Drug	[68]
**T5**	Lovastatin hydroxy acid	Drug	[69]
**T3**	D-(-)-penicillamine	Drug	—
**T3**	Tropine	Drug	—
**T3**	Heliotrine	Drug	—
**T3**	Diethyltoluamide	Insect-repellent	[70]

* A factorial experimental design included carbon source (starch) frequent addition on daily basis (once, twice, triple) and C/N ratio (10, 15, 20). T1; once C/N 10, T2; once C/N 15, T3; once C/N 20, T4; twice C/N 10, T5; twice C/N 15, T6; twice C/N 20, T7; triple C/N 10, T8; triple C/N 15, T9; triple C/N 20.

### 2.3. Bacterial composition and diversity in shrimp intestine

#### 2.3.1. Community indices

Estimation of phylotype richness was calculated according to the bias-corrected Chao1 estimator. Chao1 score showed the highest value at T7. The bacterial α-diversity was higher at T4, T6, and T7. These implied that bacterial diversity it is not necessary a sign of healthy bacterial composition. Simpson index showed the higher value for T2 and T9 (Table 4).

**Table 4:** Bacterial diversity in shrimp *Litopeaneus vanname* gut.

Index	OnceC10	OnceC15	OnceC20	TwiceC10	TwiceC15	TwiceC20	TripleC10	TripleC15	TripleC20
**Chao1**	589	520	606	524	593	601	645	539	516
**Shanno**	3.6	3.3	3.6	4.1	3.8	4	4	3.7	3.3
**Simpson**	0.082	0.12	0.086	0.038	0.046	0.037	0.057	0.058	0.11

A factorial experimental design included carbon source (starch) frequent addition on daily basis (once, twice, triple) and C/N ratio (10, 15, 20). T1; once C/N 10, T2; once C/N 15, T3; once C/N 20, T4; twice C/N 10, T5; twice C/N 15, T6; twice C/N 20, T7; triple C/N 10, T8; triple C/N 15, T9; triple C/N 20.

#### 2.3.2. Bacterial community composition

**Fig 4:**
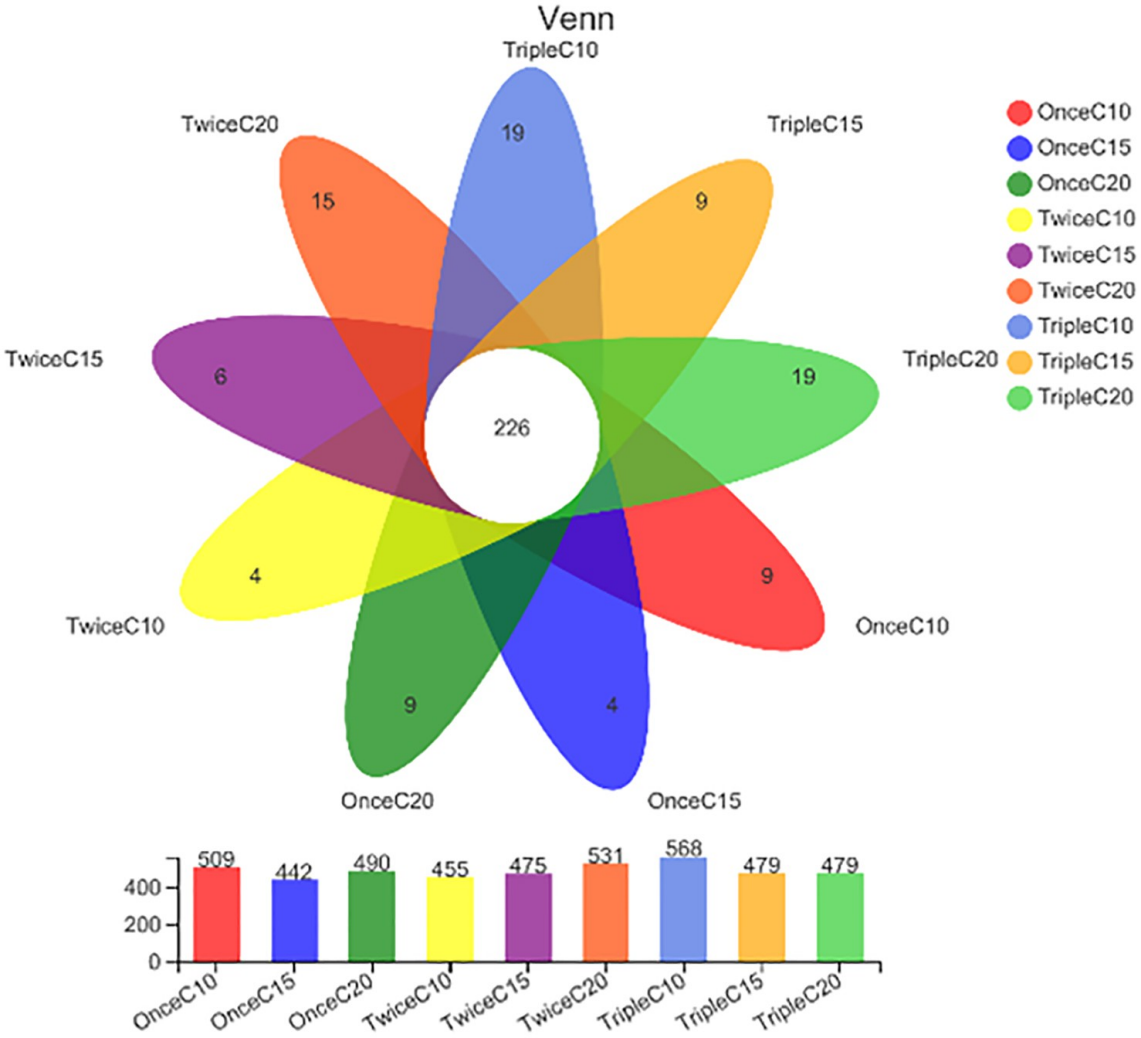
Venn diagram showing unique and shared operational taxonomic units (OTUs) of intestinal bacteria of *Litopenaeus vannamei* reared in biofloc under different frequent addition times of carbon source (starch) (once, twice, triple) on daily basis and C/N ratios (10, 15, 20). T1; once C/N 10, T2; once C/N 15, T3; once C/N 20, T4; twice C/N 10, T5; twice C/N 15, T6; twice C/N 20, T7; triple C/N 10, T8; triple C/N 15, T9; triple C/N 20.

Venn diagram analyses revealed significant differences in the frequency distribution of bacterial OTUs according to the addition frequent of starch and C/N ratio interaction. The three described treatments as they are less pathogen abundances (explained in section 3) represented differences in their bacterial OUTs count (Fig 4).

**Fig 5:**
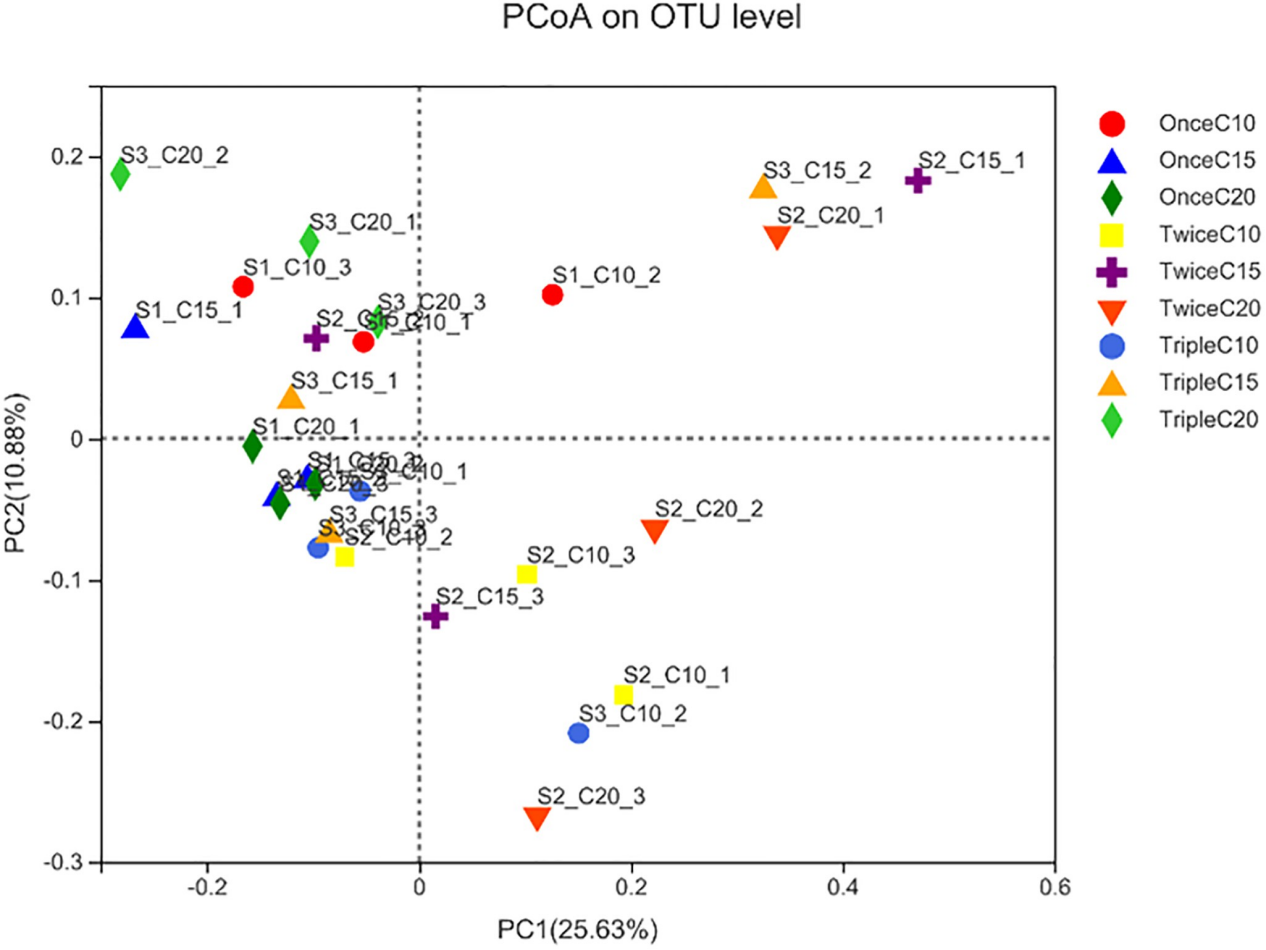
Principle coordinate analysis a (PCAa) of intestinal bacteria of *Litopenaeus vannamei* reared in biofloc under different frequent addition times of carbon source (starch) (once, twice, triple) on daily basis and C/N ratios (10, 15, 20). T1; once C/N 10, T2; once C/N 15, T3; once C/N 20, T4; twice C/N 10, T5; twice C/N 15, T6; twice C/N 20, T7; triple C/N 10, T8; triple C/N 15, T9; triple C/N 20.

Principle coordinate analysis (PCoA) showed the distance between treatments, the treatments were presented in groups depends on the distances among treatments as following: T1 and T9; T4 and T6; T2, T3, T7, and T8. In fact, T9 showed a similar distance to the T2 and T3 as they have the least pathogen abundances (section 3.2) (Fig 5).

**Fig 6:**
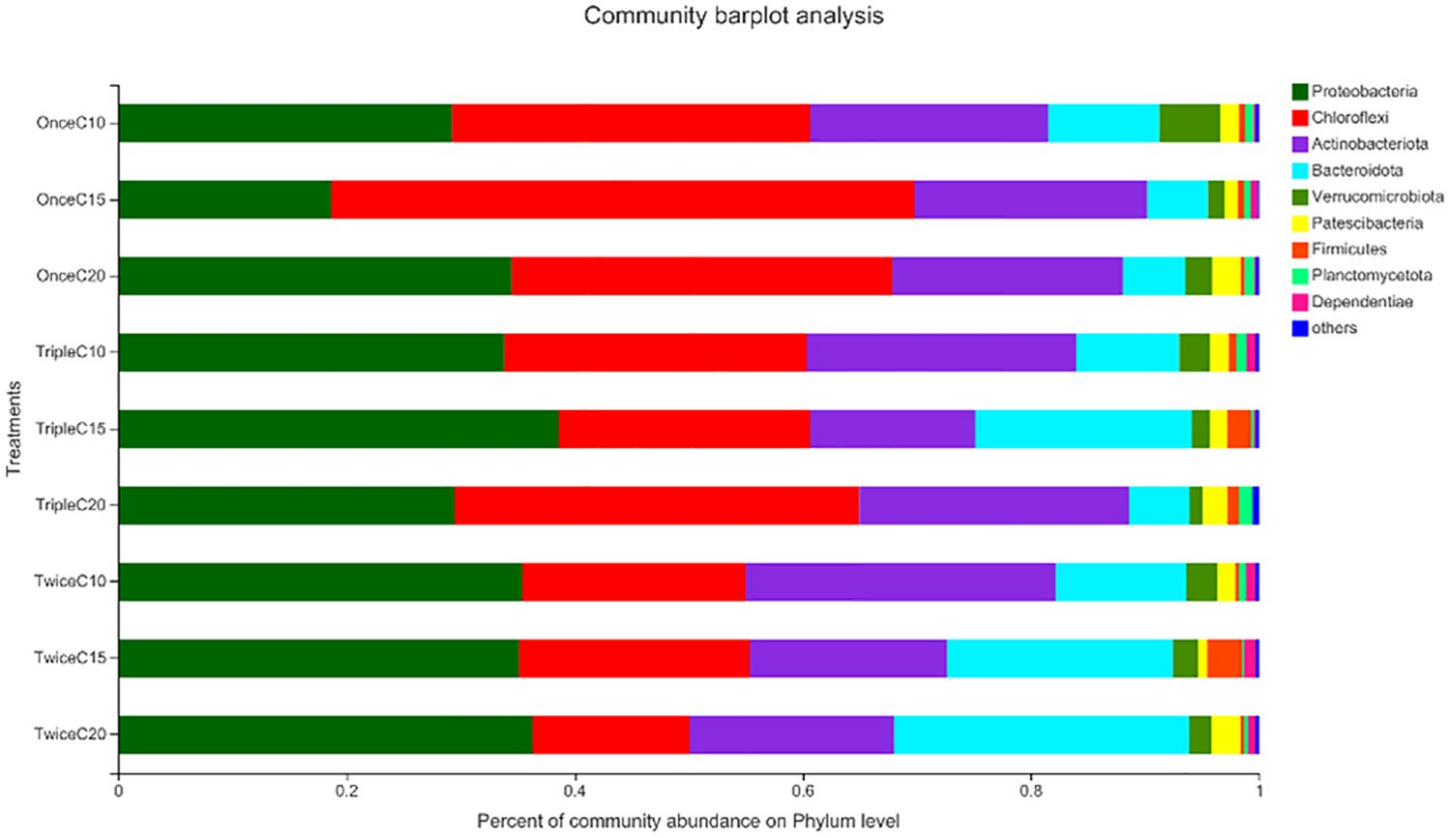
Relative abundance of the most prevalent bacterial phyla identified in the intestine of *Litopenaeus vannamei* reared in biofloc under different frequent addition times of carbon source (starch) (once, twice, triple) on daily basis and C/N ratios (10, 15, 20). T1; once C/N 10, T2; once C/N 15, T3; once C/N 20, T4; twice C/N 10, T5; twice C/N 15, T6; twice C/N 20, T7; triple C/N 10, T8; triple C/N 15, T9; triple C/N 20.

At phylum level, Treatments T2, T3, and T9 showed the least abundance level of unfavorable bacteria of Bacteroidota, whereas T2 showed the least bacterial abundance of unfavorable bacteria of Proteobacteria (Fig 6, Table 5).

**Table 5:** Relative abundance of the most prevalent bacterial phyla identified in the intestine of *Litopenaeus vannamei* reared in biofloc.

Phylum	OnceC10	OnceC15	OnceC20	TwiceC10	TwiceC15	TwiceC20	TripleC10	TripleC15	TripleC20
**Proteobacteria**	0.29	0.18	0.34	0.35	0.35	0.36	0.33	0.38	0.29
**Chloroflexi**	0.31	0.51	0.33	0.19	0.2	0.13	0.26	0.22	0.35
**Actinobacteroita**	0.2	0.2	0.2	0.27	0.17	0.17	0.23	0.14	0.23
**Bacteroidota**	0.097	0.052	0.055	0.11	0.19	0.25	0.09	0.18	0.05
**Verrucomicrobiota**	0.053	0.015	0.023	0.027	0.022	0.019	0.026	0.016	0.011
**Patescibacteria**	0.015	0.011	0.025	0.015	0.008	0.025	0.0069	0.021	0.0097
**Firmicutes**	0.0057	0.0059	0.0034	0.0028	0.03	0.0035	0.0069	0.021	0.009
**Planctomycetota**	0.0069	0.0052	0.0086	0.0062	0.002	0.0036	0.0084	0.0025	0.011
**Depndentiae**	0.0022	0.0062	0.0013	0.008	0.01	0.0057	0.0082	0.0018	0.0006

A factorial experimental design included carbon source (starch) frequent addition on daily basis (once, twice, triple) and C/N ratio (10, 15, 20). T1; once C/N 10, T2; once C/N 15, T3; once C/N 20, T4; twice C/N 10, T5; twice C/N 15, T6; twice C/N 20, T7; triple C/N 10, T8; triple C/N 15, T9; triple C/N 20.

**Fig 7:**
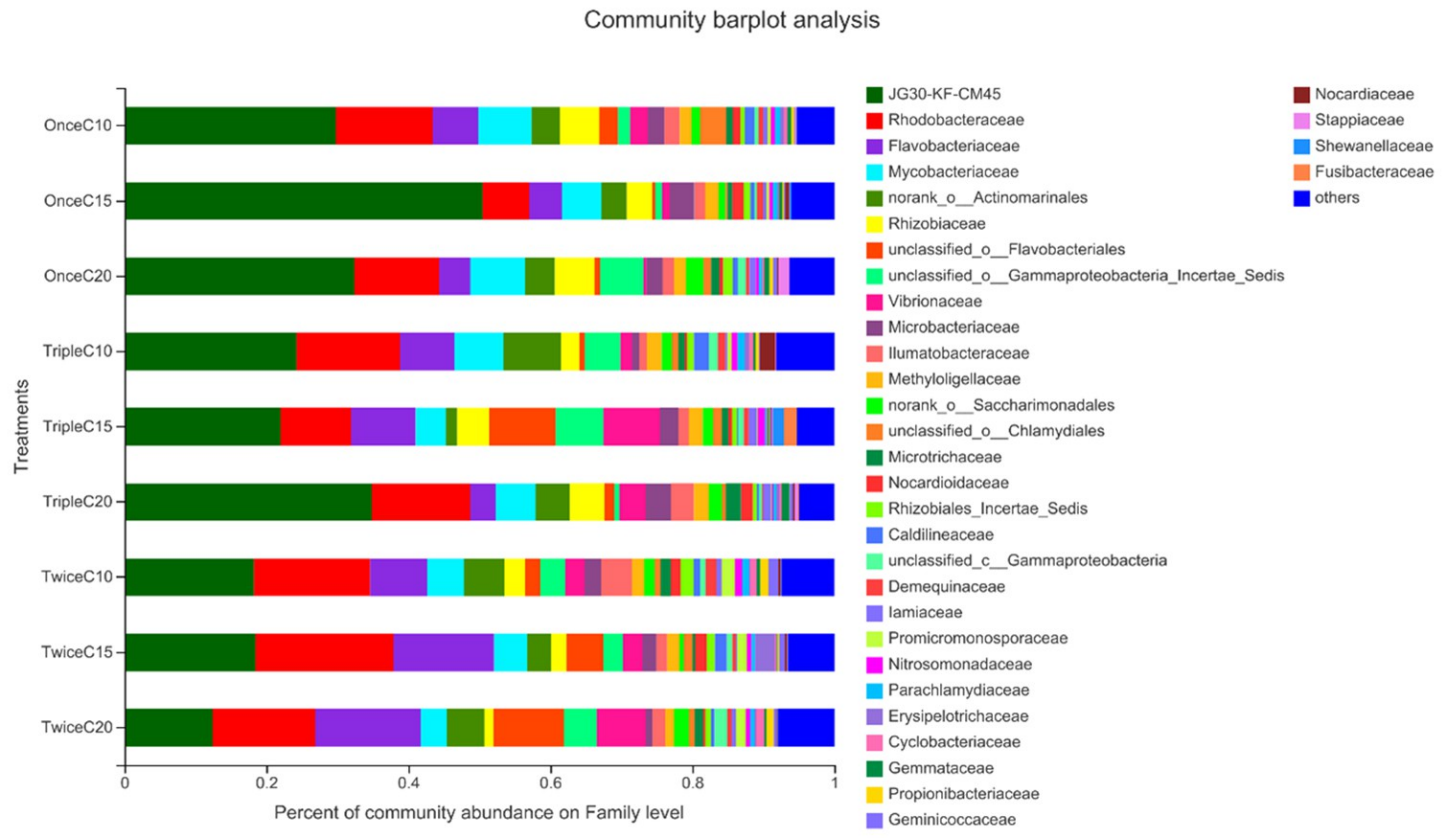
Relative abundance of the most prevalent bacterial family identified in the intestine of *Litopenaeus vannamei* reared in biofloc under different frequent addition times of carbon source (starch) (once, twice, triple) on daily basis and C/N ratios (10, 15, 20). T1; once C/N 10, T2; once C/N 15, T3; once C/N 20, T4; twice C/N 10, T5; twice C/N 15, T6; twice C/N 20, T7; triple C/N 10, T8; triple C/N 15, T9; triple C/N 20.

At family level, T2 showed the least abundance of unfavorable bacteria including Flavobacteriales and Gammaproteobacteria, whereas T3 showed the least abundance of unfavorable bacteria including Flavobacteriales and Shewanellaceae, in addition to the least abundance of pathogenic Vibrionaceae and highest abundance of beneficial bacteria of Stappiaceae and Fusibacteraceae. T9 showed the least abundance of unfavorable bacteria of Flavobacteriaceae and Gammaproteobacteria. Noteworthy, T7 was the only treatment showed the abundance of pathogenic bacteria of Nocardiaceae (Fig 7, Table 6).

**Table 6:** Relative abundance of the most prevalent bacterial families identified in the intestine of *Litopenaeus vannamei* reared in biofloc.

Family	OnceC10	OnceC15	OnceC20	TwiceC10	TwiceC15	TwiceC20	TripleC10	TripleC15	TripleC20
**JG30-KF-CM 45**	0.29	0.5	0.32	0.18	0.18	0.12	0.24	0.21	0.34
**Rhodobacteraceae**	0.13	0.065	0.119	0.16	0.19	0.14	0.14	0.09	0.13
**Flavobacteriaceae**	0.064	0.045	0.043	0.08	0.14	0.14	0.076	0.091	0.035
**Mycobacteriaceae**	0.075	0.056	0.077	0.05	0.046	0.036	0.068	0.042	0.056
**Actinomarinales**	0.039	0.035	0.042	0.056	0.033	0.053	0.081	0.015	0.048
**Rhizobiaceae**	0.055	0.035	0.055	0.029	0.022	0.012	0.025	0.045	0.048
**Flavobacteriales**	0.026	0.004	0.007	0.021	0.051	0.099	0.007	0.093	0.013
**Gammaproteobacteria**	0.016	0.009	0.061	0.035	0.027	0.046	0.05	0.067	0.007
**Vibrionaceae**	0.025	0.01	0.004	0.026	0.027	0.068	0.016	0.078	0.037
**Nocardiaceae**	0.0	0.0	0.0	0.0	0.0	0.0	0.021	0.0	0.0
**Stappiaceae**	0.0	0.0	0.016	0.0	0.0	0.0	0.0	0.0	0.0
**Fusibacteraceae**	0.0	0.0	0.016	0.0	0.0	0.0	0.0	0.017	0.0
**Shewanellaceae**	0.0	0.0	0.016	0.0	0.0	0.0	0.0	0.016	0.0

A factorial experimental design included carbon source (starch) frequent addition on daily basis (once, twice, triple) and C/N ratio (10, 15, 20). T1; once C/N 10, T2; once C/N 15, T3; once C/N 20, T4; twice C/N 10, T5; twice C/N 15, T6; twice C/N 20, T7; triple C/N 10, T8; triple C/N 15, T9; triple C/N 20.

**Fig 8:**
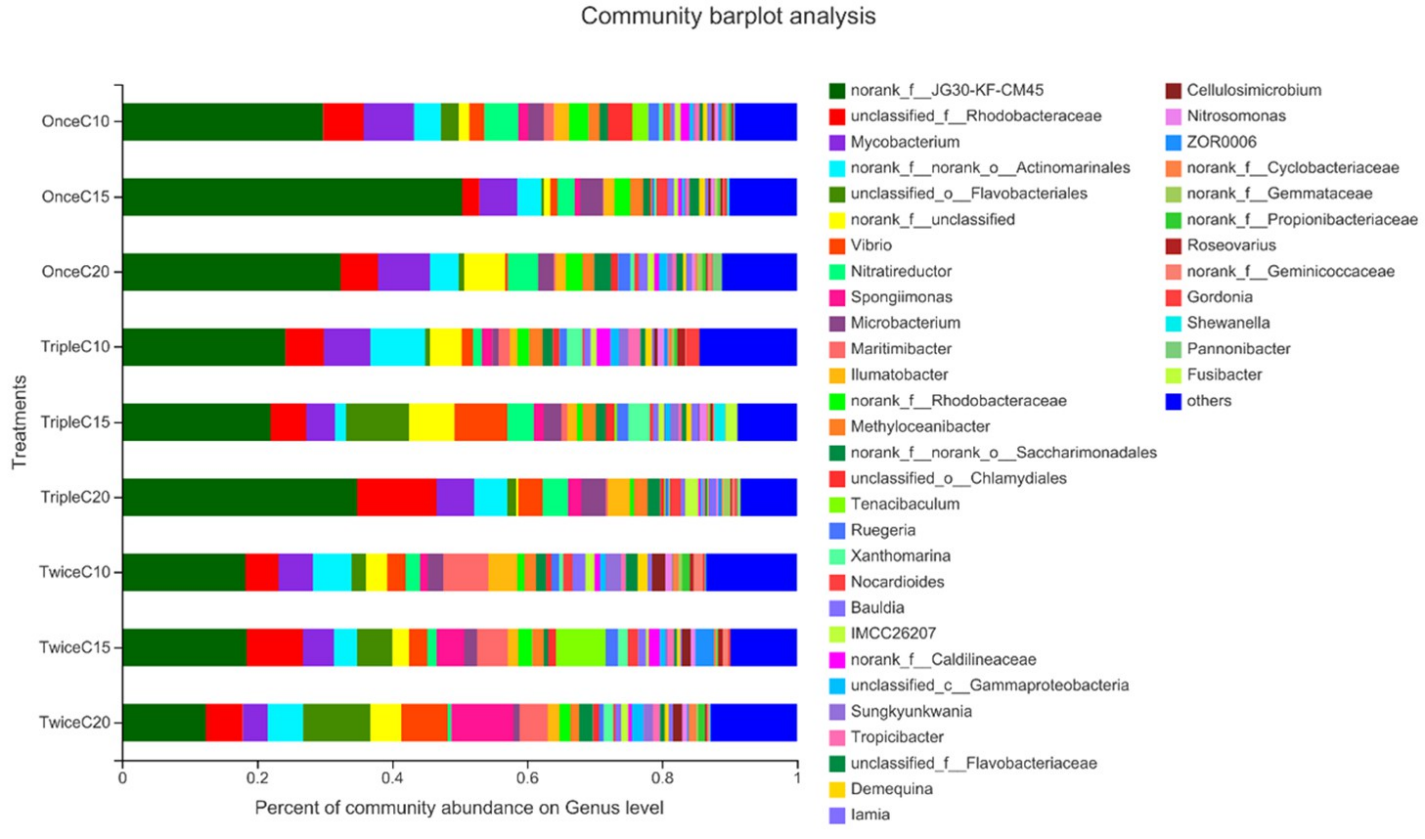
Relative abundance of the most prevalent bacterial genus identified in the intestine of *Litopenaeus vannamei* reared in biofloc under different frequent addition times of carbon source (starch) (once, twice, triple) on daily basis and C/N ratios (10, 15, 20). T1; once C/N 10, T2; once C/N 15, T3; once C/N 20, T4; twice C/N 10, T5; twice C/N 15, T6; twice C/N 20, T7; triple C/N 10, T8; triple C/N 15, T9; triple C/N 20.

At genus level, T2, T3, and T9 showed the least bacterial abundances of Tenacibaculum (pathogen) and Gammaproteobacteria (unfavorable bacteria), but T2 and T9 showed highest abundance of unfavorable bacteria of Micobacterium. T2 and T3 showed the least bacterial abundance of unfavorable bacteria of Flavobacteriales. In fact, T3 showed the least Vibrio abundance (Fig 8, Table 7).

**Table 7:** Relative abundance of the most prevalent bacterial genera identified in the intestine of *Litopenaeus vannamei* reared in biofloc.

Genus	OnceC10	OnceC15	OnceC20	TwiceC10	TwiceC15	TwiceC20	TripleC10	TripleC15	TripleC20
**JG30-KF-CM 45**	0.29	0.5	0.32	0.18	0.18	0.12	0.24	0.21	0.34
**Rhodobacteraceae**	0.059	0.024	0.055	0.049	0.083	0.054	0.056	0.053	0.11
**Mycobacterium**	0.075	0.056	0.077	0.023	0.019	0.0093	0.068	0.042	0.056
**Actinomarinales**	0.039	0.035	0.042	0.056	0.033	0.053	0.081	0.015	0.048
**Flavobacteriales**	0.026	0.004	0.007	0.021	0.051	0.099	0.007	0.093	0.013
**Vibrio**	0.022	0.01	0.0042	0.026	0.027	0.068	0.016	0.07	0.036
**Nitratireductor**	0.05	0.025	0.044	0.021	0.014	0.005	0.013	0.04	0.037
**Spengiimonas**	0.014	0.007	0.0006	0.011	0.04	0.09	0.014	0.013	0.019
**Microbacterium**	0.023	0.034	0.022	0.023	0.019	0.009	0.009	0.026	0.036
**Maritimibacter**	0.015	0.001	0.002	0.065	0.045	0.041	0.016	0.009	0.002
**Tenacibaculum**	0.023	0.0	0.0	0.0	0.073	0.0	0.0	0.004	0.002
**Gammaproteobacteria**	0.0056	0.0035	0.04	0.007	0.007	0.017	0.012	0.008	0.0039
**Cellulosimicrobium**	0.004	0.003	0.0007	0.019	0.013	0.013	0.0053	0.001	0.0
**Shewanella**	0.0	0.0	0.0	0.0	0.0	0.0	0.0	0.016	0.0

A factorial experimental design included carbon source (starch) frequent addition on daily basis (once, twice, triple) and C/N ratio (10, 15, 20). T1; once C/N 10, T2; once C/N 15, T3; once C/N 20, T4; twice C/N 10, T5; twice C/N 15, T6; twice C/N 20, T7; triple C/N 10, T8; triple C/N 15, T9; triple C/N 20.

#### 2.3.3. Bacteria and active molecules interaction

Proline was associated with Bacteroidota, Flavobacteriaceae, Gammaproteobacteria, and Flavobacteriales. Plumbagine was associated with Norcardiaceae. Phytosphingosin was associated with Bacteroidota. Phosphocholine compound was associated with Bacteroidota. The monobutyl ether, benzofuran, and piperidone were associated with Micobacteriaceae and Mycobacterium.

## 3. Discussion

Biofloc is a rich source of bioactive molecules, which their composition depends on nutrient input, water source, and bacterial composition. Three treatments (C/N 15, 20 once a day, and C/N 20 triple a day) have showed a merit over other treatments in term of low pathogenic and unfavorable bacterial abundances, and high commensal bacterial abundance. These changes could be attributed to the bioactive molecule composition as a result of bacterial composition change. The available literature revealed the possible effect of some molecules on bacterial composition, while other molecules have not been studied in a correlation with the existed bacteria in our current study. Proline, plumbagine, phytosphingosin, phosphocholine, monobutyl ether, benzofuran, and piperidone molecules were associated with different bacterial abundances.

Proline is a metabolized molecule by all organisms as a protein building block. It may also serve as a source of carbon, nitrogen, and energy source, or as an osmoprotectant in bacteria which including the Gram-negative enteric bacteria (*Escherichia coli*, *Salmonella typhimurium*) [71]. In fact, proline contributes to the pathogenesis of various pathogenic bacteria [72]. Since an interruption of proline metabolism (Δ1-pyrroline-5-carboxylate) and uptake attenuates virulence of certain pathogens [72]. In our study, T9 showed the highest proline level, and lower pathogenic abundance of Tenacibaculum, thus proline availability may indicate for the lower pathogenic bacterial abundance. On the other hand, proline amino acid also serves as a functional molecule in peptide’s structure as antimicrobial agent. A Ls-Stylicin 1, isolated from penaeid shrimp *Litopenaeus stylirostris*, has a proline rich N-terminal region, shows an effect against Gram-negative bacteria through the LPS-binding activity in an in vitro study [73, 74]. Future studies are required to investigate the proline forms including peptides and their effect on BF system biosecurity.

Phosphocholine (PC) presents more than 10% of the all bacteria as an essential cell wall phospholipid component [75], and present from zero to 73% as cell wall component in bacteria such as *Pseudomonas aeruginosa* and *Acetobacter aceti* [75, 76]. PC residues are required for the pathogenic bacteria and their biological activities which including transformability, autolysins, daughter cells separation, and anchoring a family of surface proteins. These proteins play important roles in bacterial infection including Pseudomonas, Brucella, Bartonella, Francisella, Borrelia, and Pneumococci [32, 76]. Pneumococci are unique bacteria among Gram-positive bacteria regarding to the lipoteichoic (LTA) and teichoic acid (TA) contents which they have identical chains, these chains are substituted with the PC residues [77, 78]. Autolytic enzyme, N-acetyl-muramyl-L-alanine-amidase (LytA), requires PC to be activated [32, 79]. In fact, surface-exposed PC residue can be recognized by the host’s innate defense system through the C-polysaccharide reactive protein (CRP) [32]. In this context, it is recommended for the future studies to investigate the effect of PC on host immunity, and whether the PC inhibition could be a beneficial practice in BF biosecurity control. In our study, T3, T7 and T9 treatments showed the highest relative fold-change (2.8, 2.6, 3.1, respectively) comparing to T2 treatment. This PC content may indicate for the pathogenic bacterial abundances.

Anaerobic bacteria use oligo- and poly-ethylene glycols as carbon source, and polyethylene glycol monomers can be metabolized to polyglycol molecules which reduced by one glycol unit in bacteria [80–83]. Triethylene Glycol (TEG) promotes the growth of pathogenic bacteria by up-regulating virulence-associated genes and proteins such as in Streptococcus mutan [83]. These genes are contributing to the polysaccharide synthesis in bacterial biofilms [83]. TEG penetrates the biofilms’ exopolysaccharide matrix which activates the TCSTs via vicRK signaling pathway [83]. In our study, T6 showed the highest TEG relative fold change along with the highest bacterial abundances of Vibrio, Bacteroidota, and Flavobacteriales. It is a possible practice that applying a chelating agent to the BF could decrease TEG and the abundance of undesired bacteria.

Plumbagin is a secondary plant metabolite exhibiting a toxicity against several pathogenic and non-pathogenic microbes [84, 85], for example, it inhibits the proliferation of Mycobacterium, Pseudomonas aeruginosa, Bacillus subtilis, Staphylococcus aureus, and Proteus vulgaris [49, 85]. However, the antimicrobial effect is restricted to a few bacterial species. In fact, some bacteria were growing in its presence such as *Escherichia coli* and *Salmonella typhimurium* [49]. Plumbagin inhibits cytokinesis in Bacillus subtilis via FtsZ assembly inhibition. This FtsZ assembly has a key role in construction of the cytokinetic-ring which mediates the bacterial cell division. Plumbagin binds to BsFtsZ and reduces the assembly and GTPase activity of BsFtsZ [49]. In our study, T9 showed the highest level of plumbagine, but a highest abundance of Mycobacterium. This may be explained by the insufficient concentration of plumbagine against Mycobacterium.

Sphingolipid occur in cellular membranes of all eukaryotes, but only a few bacterial genera have it in their cell membrane [86–88]. Sphingolipid shows antimicrobial activity against a range of gram-positive and gram-negative pathogenic bacteria including *Pseudomonas aeruginosa* and *Escherichia coli* [50]. In our study, T9 showed the highest level of phytosphingosin and lower abundance level of Tenacibaculum. But the T2 T3 treatments also showed the lower abundance level of Tenacibaculum. These could reveal a multi factors affecting this bacterial abundance in BF system.

Benzofurans are a class of heterocyclic compounds which are sourced from plants and synthetic compounds [60]. These molecules exhibit an antimicrobial activity against some bacteria including *Mycobacterium tuberculosis*, *Klebsiella pneumoniae*, *Pseudomonas aeruginosa*, *Escherichia coli*, *Staphylococcus aureus*, *Bacillus subtilis*, *Bacillus megaterium*, *and Sarcina lutea* [60, 89–91]. The antimicrobial activity depends on the substitution at the heterocyclic furan ring than on substitution at the aromatic moiety [92]. In our study, T3 showed the highest level of benzofuran and lower bacterial abundance of Tenacibaculum, Vibrio, and Vibrionaceae. This could imply the effect of benzofuran against pathogenic bacteria. But the T3 showed also the highest bacterial abundance of Mycobacterium, which can be explained by the insufficient level of benzofuran against this bacterial abundance.

In BF system, heterotrophic bacteria assimilate the ammonia which resulting from the degraded uneaten feed and feces particles, or the ammonia excretion of aquatic animals [16]. Supplying carbohydrates increases the heterotrophic bacterial activity and changes the bacterial composition in BF system [16]. High C/N ratios can lead to nitrate reduction to ammonium, while low C/N ratios can lead to an inhibition of denitrification process [24]. For an example, C/N ratio ranged from 9:1 to 12:1 induces nitrification process, while C/N ratio ranged from 15:1 to 18:1 induces heterotrophic activity [25]. However, C/N 20 allows the accumulation of nitrite and nitrate [93]. In our study, T2, T3 and T9 treatments showed the lower abundance of both pathogenic (Tenacibaculum) and unfavorable (Bateroidota, Flavobacteriaceae) bacteria, in addition, T2 and T9 treatments showed the lower Gammaproteobacteria abundance. Thus, C/N ratio (15–20) with frequent addition once a day or C/N ratio (20) with frequent addition triple a day may increase the BF biosecurity level. Based on Vibrio abundance, C/N 20 with frequent addition once a day (T3) showed the lower bacterial abundance, in addition, T3 was the only treatment which showed an abundance of beneficial bacteria (Stappiaceae). As it is difficult to draw a conclusion among treatments based on the unfavorable and pathogenic bacterial abundances, it is recommended for the future studies to investigate the immune response in the cultured species.

Natural products play an important role in chemical biology and nomination of therapeutics agent, which need further investigation to approve its safety application [60]. BF is a complicated ecosystem regarding to its bacterial complexity, continuous nutrient input, and less nutrient output, this provided an internal dependent component in BF system, that modulating those components could achieve a high level of environmental biosecurity control and aquatic productivity achievement. This study revealed the associated bioactive molecules to the bacterial abundances, which could reveal the applicable potence of those molecules as biosecurity agents in a BF or any other aquaculture systems. Those molecules can be divided to bacterial activities’ inducers or inhibitors. It is recommended for the future studies to investigate the potence of those molecules individually and collectively on the biosecurity control in aquaculture systems.

## 4. Materials and methods

### 4.1. Ethics protocol and experimental design

The experiment was conducted strictly under the research protocols which approved by Yellow sea fisheries research institute, Chinese academy of fishery sciences.

**Fig 9:**
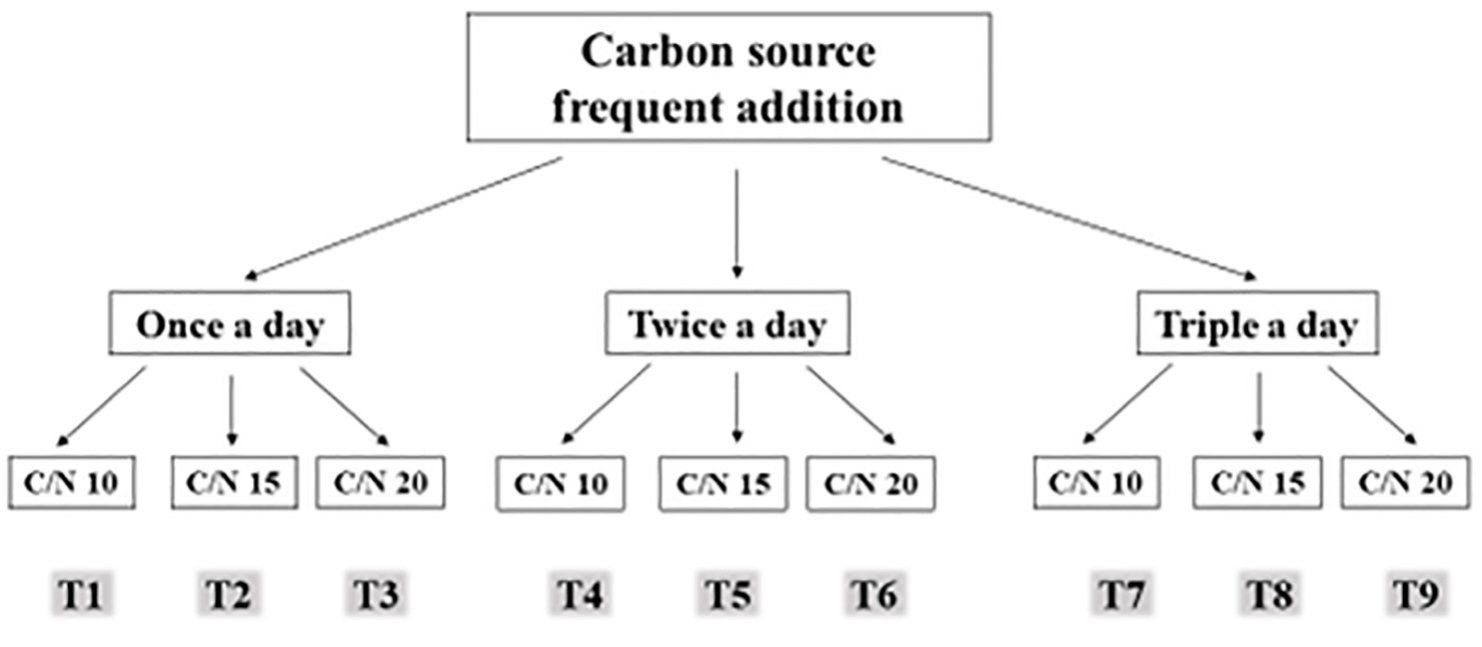
Experimental design. A factorial design with nine treatments. The effect of carbon source (starch) frequent addition (once, twice, triple) at daily basis, and C/N ratio (10, 15, 20) interaction on shrimp *Litopeaneus vanname* gut microbiota composition.

BF started up with the addition of maize starch as carbon source to the aquaculture water in a ratio with feed nitrogen content. The appropriate C/N ratio induces the activity of heterotrophic bacteria assimilating nitrogen wastes in the aquatic environment. But the nutrient availability forms the microbial structure and subsequently the flocs level and their content of non-viable bacterial content. A factorial experimental design included carbon source (starch) frequent addition on daily basis (once, twice, triple) and C/N ratio (10, 15, 20). T1; once C/N 10, T2; once C/N 15, T3; once C/N 20, T4; twice C/N 10, T5; twice C/N 15, T6; twice C/N 20, T7; triple C/N 10, T8; triple C/N 15, T9; triple C/N 20 (Fig 9).

Feeding rate was 5% of total biomass of each aquaria unit per day, feeding regime was 3 times per day (8:00, 12:00, 4:00). A commercial diet was used for shrimp feeding (crude protein; 41%, crude fat; 4%, crude fiber; 6%, ash; 15%, lysine; 2%, total phosphorus; 0.8%, moisture; 12%) (Alpha feed, Huai’an, Jiangsu, China) for the experimental period of 45 days. The carbon source was added after half an hour of first feeding (8:30) on daily basis. A total of 486 shrimp *Litopenaeus vannamei* (1.3±0.3 g) were divided among treatments units (18 shrimp/aquaria).

The GC/LC and high-throughput sequencing analysis were performed for the BF water and shrimp intestinal bacterial community, respectively, after two months of experimental period. The water quality was within the normal range (oxygen; 5.6±0.3 mgL^-1^, salinity; 29±1 ppt L^-1^, pH; 6.6±0.5, NH^4+^; 0.02–0.028 mgL^-1^, NO^2-^; 0.07 ± 0.4 mgL^-1^, NO3-; 0.64 ± 0.6 mgL^-1^) [94].

### 4.2. Water quality analysis

Water temperature, dissolved oxygen, salinity and pH were measured by YSI Incorporated device (Yellow Springs, OH, USA). Ammonia-N, nitrate-N, and nitrite-N were measured using a QuAAtro nutrient auto analyzer (Seal Analytical Ltd., Germany). The concentrations of three dissolved inorganic nitrogen (DIN) were measured using in suit water quality nutrient analyzer (SINOHLK-NutriS, Xiamen, China).

### 4.3. Extraction of metabolites

The non-targeted metabolites analysis was conducted according to the method of [95] with some changes. Briefly, supernatant of each filtrated (0.22 μm filter paper) biofloc water sample (50 ml) was thawed and mixed with 400 μL of pre-cold methanol/acetonitrile/aqueous solution (2:2:2, v/v) and vortexed (Haimen City Qilin Medical Instrument Factory, Haimen, Jiangsu, China) for 60 s before centrifuged (Xiangyi Centrifuge Instrument Co., LTD., Changsha, Hunan, China) at 14000 g under -20°C for 20 min. Subsequently, the supernatant was vacuum dried using a vacuum concentrator (Eppendorf LTD., Hamburg, Germany) and reconstituted in 100 μL of acetonitrile aqueous solution (acetonitrile-water = 1:1, v/v) and vortexed for 60 s before centrifuged at 14000 g under 4°C for 15 min. The resulted mixture was filtered through 0.22 μm syringe filter and used for LC-MS/MS analysis. All the samples were performed in three biological replicates.

### 4.4. LC-MS/MS condition

Separation of the metabolites was accomplished by Agilent 1290 Infinity LC Ultra-performance liquid chromatography (UHPLC) (Agilent Technologies, Santa Clara, CA, USA), coupled to a quadrupole time-of-flight (AB Sciex TripleTOF 6600), and equipped with an HILIC column for separation (1.7 μm, 100 × 2.1 mm, Waters ACQUITY UPLC BEH Amide). The temperature of the column was maintained at 25°C, while the autosampler was set at 4°C. The mobile phase was carried out as follows: (A) water + 25 mM ammonium acetate + 25 m ammonia, and (B) acetonitrile. The flow rate was set at 0.5 mL min−1 and 2 μL of each sample was injected after equilibration. 20 μL of filtrate from each sample was mixed to make the quality control (QC) samples to monitor deviations of the analytical results and compare them to the errors caused by the analytical instrument itself.

The elution program was used as follows: the gradient elution procedure was as follows: 0–0.5 min, 95% B; 0.5–7 min, B from 95% linear change to 65%; 7–8 min, B from 65% linear change to 40%; 8–9 min, B maintained at 40%; 9–9.1 min, B from 40% linear change to 95%; 9.1–12 min, B maintained at 95%. The column eluent was infused into a Thermo Q Exactive mass spectrometer (Thermo Fisher Scientific, Waltham, MA, USA) with a spray voltage of 5.5 kV in positive and negative modes. The ESI source conditions after HILIC chromatographic separation are as follows: Ion Source Gas1 (Gas1): 60, Ion Source Gas2 (Gas2): 60, Curtain gas (CUR): 30. The capillary temperature was 600°C. TOF MS scan m/z range: 60–1000 Da, product ion scan m/z range: 25–1000 Da, TOF MS scan accumulation time was 0.20 s/spectra, product ion scan accumulation time was 0.05 s/spectra; Secondary mass spectrometry information dependent acquisition (IDA) was obtained, and the high sensitivity mode was set, Declustering potential (UU dp) was ± 60 V (plus or minus two modes), Collision Energy was 35 ±15 eV, IDA was set as follows Exclude isotopes within 4 Da, Candidate ions to monitor per cycle was10.

### 4.5. High-throughput sequencing of bacterial intestinal community

The total DNA of full intestine samples were extracted using the TIANamp Bacteria DNA Kit (Tiangen Biotech, Beijing, China), the temperature cycling conditions for PCR were as following: the initial heating was at 95°C for 5 minutes, followed by 30 cycles at 94°C for 30 s, 50°C for 30 s, 72°C for 45 s and a final extension at 72°C for 10 min. DNA integrity was confirmed by agarose gel electrophoresis. Using NanoDrop Spectrophotometer (Thermo Scientific, USA), the bacterial DNA concentration was measured. The V3-V4 region of 16SrRNA gene, a specific conserved sequence region of bacterial DNA, with the primers 338F (5’-ACTCCTACGGGAGGCAGCAG-3’) and 806R (5’-GGACTACHVGGGTWTCTAAT-3’) (Xu, 2016 #537) was amplified by polymerase chain reaction using MyCyclerTM thermal cycler (BIO-RAD, USA). The bacterial DNA was purified and sequenced by Illumina-Miseq by Majorbio.

### 4.6. Statistical analysis

The UHPLC data were normalized to a total peak intensity, the processed data were analyzed by R package (ropls), where it was subjected to multivariate data analysis, including Pareto-scaled principal component analysis (PCA) and orthogonal partial least-squares discriminant analysis (OPLS-DA). The 7-fold cross-validation and response permutation testing were used to evaluate the robustness of the model. The variable importance in the projection (VIP) value of each variable in the OPLS-DA model was calculated to indicate its contribution to the classification. Metabolites with the VIP value >1 was further applied to Student’s t-test at univariate level to measure the significance of each metabolite, the P values less than 0.05 were considered as statistically significant.

High-throughput sequencing Paired-end (PE) reads were spliced by FLASH software [96] software according to the overlap relationship, and Fastp [97] software was used for quality control and filtration of original sequencing sequences. After data optimization, using UPARSE [98] software for OTU clustering and statistical analysis of biological information for the sequence according to the similarity of 97% [98, 99]. The RDP Classifier [100] software was used for species classification analysis for each sequence. According to the results of taxonomic analysis, the community structure of the samples at different classification levels was measured by statistical analysis. The Alpha diversity was calculated using MOTHUR [101]. The SPSS Statistics 22 was used for statistical analysis of data differences, when value of P<0.05 was considered as significant and *P*<0.01 was considered as extremely significant [102].

## 5. Conclusions

Biofloc is a rich source of bioactive molecules, which their composition depends on nutrient a viability and subsequently the bacterial composition. Proline, plumbagine, phytosphingosin, phosphocholine, monobutyl ether, benzofuran, and piperidone molecules were associated with different bacterial abundances. These molecules are nominated as biosecurity agents to control the biosecurity level in BF system. Based on available literature, other molecules have not been investigated with correlation to the presented bacteria in this study, which they could have novel agents in biosecurity control. This information could provide applicable feed’s or water’s additives achieving higher biosecurity control in aquaculture systems.
